# The Development and Preliminary Application of the Chinese Version of the COVID-19 Vaccine Literacy Scale

**DOI:** 10.3390/ijerph192013601

**Published:** 2022-10-20

**Authors:** Yihui Li, Yi Guo, Xusheng Wu, Qingyuan Hu, Dehua Hu

**Affiliations:** 1 Department of Biomedical Informatics, School of Life Sciences, Central South University, Changsha 410013, China; 2 Shenzhen Health Development Research and Data Management Center, Shenzhen 518000, China; 3 Xiangya III Hospital, Central South University, Changsha 410013, China

**Keywords:** COVID-19, vaccine literacy, scale, vaccination, Chinese, general public

## Abstract

(1) Background: Vaccine literacy (VL) of the public is crucial to deal with anti-vaccination rhetoric. This study aims to (1) develop a Chinese COVID-19 Vaccine Literacy Scale and examine the factor structure and psychometric characteristics, and (2) explore the association between COVID-19 VL and sociodemographic characteristics and other variables; (2) Methods: An online cross-sectional survey was conducted among 362 Chinese residents from 23 May 2022 to 31 May 2022 using snowball sampling; (3) Results: Exploratory and confirmatory factor analysis indicated that the scale of 15 items, consisting of three factors, functional, interactive and critical vaccine literacy, explained 63.3% of the total variance. Cronbach’s α coefficient was 0.885 for the overall scale: 0.838, 0.891, and 0.857 for three subscales, respectively. The results showed a medium level of vaccine literacy (M = 3.71, SD = 0.72) and significant differences among functional, interactive, and critical vaccine literacy (*p* < 0.001). The level of vaccine literacy grew with the level of education (*p* < 0.001) and age (*p* = 0.049). Men, participants who were single, or those living in rural areas had a lower level of vaccine literacy; (4) Conclusions: The Chinese COVID-19 VL Scale has adequate validity and reliability for assessing vaccine literacy among Chinese residents. A deep understanding of the factors that affect vaccine literacy is needed.

## 1. Introduction

COVID-19 is a major public health challenge. The World Health Organization officially defined it as a pandemic due to its severity and transmissibility in 2020 [1]. Since its emergence in 2019, COVID-19 has been sweeping people around the world, with huge negative impacts on people’s lives and health [2,3,4,5,6,7,8], economic activities [9,10,11], and lifestyles [12,13,14,15,16,17]. With the emergence of Omicron variants, the number of COVID-19 infections is still rising. As of 8 August 2022, there have been 581,686,197 confirmed COVID-19 infections and more than 6.4 million deaths worldwide [18]. As a member of a community with a shared future for mankind, China is also experiencing the trauma caused by the pandemic. In the face of such repeated epidemic, regular vaccination is by far the most effective means for the regular epidemic prevention and control of COVID-19. Since January 2022, China has also begun to advocate a new booster vaccination round of COVID-19 vaccines for all people [19]. How to effectively implement nationwide vaccination and carry out precise prevention and control is a major issue.

However, with the emergence of the infodemic, a large number of anti-vaccination rhetoric has emerged [20]. Vast quantities of false information has flood the Internet, especially in the field of health and hygiene [21], which was characterized by fast dissemination. Some studies have pointed out that anti-vaccination remarks were more likely to spread on the Internet than those supporting vaccination [22]. Since it was difficult for the general public to distinguish between rumors and objective facts, such information may have an adverse impact on the prevention and control of COVID-19 [23]. How to fight the infodemic has become a major problem in the field of public health [24]. In this context, in addition to our health and economic systems, public health literacy is of particular importance [25]. Previous studies have also shown a positive relationship between health literacy and COVID-19 vaccine acceptance [26].

Vaccine literacy was proposed based on this context, which was based on the concept of health literacy; Ratzan defined it as “not just knowledge about vaccines, it is closer to the development of a reduced complexity system that can be used by people to communicate and provide vaccine-related information, which is the premise and foundation of the stable and orderly operation of the health system. This system aims to strengthen the social norms of vaccination and provide a foundation of vaccine literacy appropriate to age, mind, gender, and environment for achieving herd immunity” [27]. Some scholars have pointed out that different from the concept of health literacy, vaccine literacy was more targeted for vaccination [28]. Vaccine literacy referred to “people’s knowledge, motivation and ability to discover, understand and use information to make vaccination decisions”. Several studies have shown a significant association between vaccine literacy and vaccination [29,30,31]. Therefore, it is critical to develop a COVID-19 vaccine literacy scale for the general population to assess people’s ability to collect and understand information about vaccination to implement targeted interventions for vaccination.

At present, the relatively mature scale applied abroad is derived from the Ishikawa Test [32], a health literacy self-assessment questionnaire for patients with chronic diseases, which is based on the three-level health literacy model proposed by Nutbeam [33]. A total of 14 items were designed to examine the health literacy of patients with type 2 diabetes at three levels: functional, interactive and critical health literacy. Biasio et al. [28] developed Health Literacy about Vaccination in adulthood in Italian (HLVa-IT) based on the Ishikawa test and applied it to the Italian adult population. The scale has been translated into multiple languages and used in various studies, with appropriate statement adaptation and cultural tuning for specific vaccine types and national cultural background, such as Thai [34], Turkish [35], Spanish [36], etc., all of which showed good psychometric characteristics.

However, there has been scarce research to date on the VL of the Chinese population and the Chinese version of the COVID-19 vaccine literacy scale has not been developed and validated. Only two studies have been performed with this aim, one of which was related to a specific vaccine (HPV vaccine) and only examined female college students’ knowledge of HPV vaccine [37] and the other examined parents’ vaccine literacy in the context of a specific vaccine scandal [38], but the set of test items were too few and limited to a certain event background; it was not generalizable. Therefore, the purpose of this study is to: (1) Translate the English version of the COVID-19 vaccine literacy scale compiled by Italian scholar Biasio based on the three-level model of health literacy of Nutbeam (2000), and appropriate supplements and revisions are made in combination with local culture to develop a COVID-19 vaccine literacy scale suitable for Chinese residents. A pilot study will be conducted to test the factor structure and psychometric characteristics of the scale, providing a theoretical basis for scientific and objective assessment of the vaccine literacy level of the Chinese public; and (2) Explore the association between COVID-19 VL and sociodemographic characteristics and other variables so as to carry out targeted vaccination campaigns for people with low vaccine literacy and promote pandemic prevention and control.

## 2. Materials and Methods

### 2.1. Study Design

On the basis of literature research and expert consultation, this study took Nutbeam’s (2000) three-level model of health literacy as the theoretical basis [39], and translated the scale of Health Literacy about Vaccination in adulthood in Italian (HLVa-IT) authorized by Italian scholar Biasio [28] in accordance with the scale’s cross-cultural translation adjustment guide [40]. Based on China’s cultural background and language habits, two additional items on interactive vaccine literacy were added to the original scale (“Did you know where to get information about the COVID-19 vaccine?” “Did you or would you articulate the COVID-19 vaccine information you want?”) to form the initial version of the COVID-19 vaccine literacy scale for Chinese residents (see Appendix A). The questionnaire consisted of two parts: the basic information of the participants and the COVID-19 Vaccine Literacy Scale.

The basic information of the participants included the following: gender, age, education, permanent residence, marital status, occupation, and average monthly income; vaccination status, history of other vaccinations, and sources of COVID-19 vaccine information. The initial version of the COVID-19 Vaccine Literacy Scale consisted of 14 items of HLVa-IT and 2 items supplemented based on domestic and foreign literature, including 3 dimensions: functional vaccine literacy (5 items), interactive vaccine literacy (7 items) and critical vaccine literacy (4 items). Functional vaccine literacy aims to assess people’s basic reading and writing skills. Interactive vaccine literacy focuses on assessing individual skills, which refer to higher-level cognition, literacy, and social skills that enable people to extract valuable and meaningful information from different exchanges of information in daily life and apply it to changing circumstances. Critical vaccine literacy is the empowerment of individuals and communities. It is the highest level of cognitive and social skills which aims to assess whether people can evaluate the information they have obtained and use it to solve problems and make decisions.

A five-point Likert scale was used to rate the responses to the items. The functional vaccine literacy component was scored in a reverse way (5—none; 4—less; 3—once in a while; 2—often; 1—always), whereas the interactive and critical vaccine literacy sections (1—none; 2—less; 3—once in a while; 4—often; 5—always) used positive scoring. The HLVa-IT questionnaire, the main body of the scale, has been used to measure the level of COVID-19 vaccine literacy in previous studies and has been verified to have good psychometric characteristics in studies conducted in different countries [29,34,35,41,42], which can be used for the subjective measurement of vaccine literacy levels in individuals and groups.

### 2.2. Data Collection

A cross-sectional Survey was performed in this study, collecting data through online platforms from 23 May 2022 to 31 May 2022. The questionnaire was prepared by the software of “Wen Juan Wang” (https://www.wenjuan.com/ (accessed on 23 May 2022)) and forwarded to the subjects in the form of links and QR codes through social software, such as Weibo, WeChat and QQ. Using the snowball sampling method, participants were first recruited randomly online and requested to spread the questionnaires to other eligible participants. To ensure the quality of the data, the survey allowed only one response per social media account and device. Inclusion criteria for this study were: (1) living in mainland China for more than six months; (2) adults over 18 years of age; (3) informed consent and voluntary participation in this study. Exclusion criteria were: (1) the time spent answering the questionnaire was less than 90 s; (2) choosing a city other than Beijing as the capital of China; (3) suffering from mental illness or cognitive impairment; and (4) refusing to participate in this study.

Before the formal investigation, a pre-survey was conducted on 30 subjects to ensure that there would be no ambiguity or difficulty in answering the questionnaire when the participants read, understand and answer the items. The vast majority of participants indicated that the length of the questionnaire was reasonable and the question was easy to understand.

A total of 28 measurement items were included in this study, and a formal sampling survey was conducted according to the principle that the sample size is 5–10 times the number of items on the scale [43]. A total of 401 questionnaires were distributed, 39 invalid questionnaires were excluded, and 362 valid questionnaires were finally recovered, with a valid recovery rate of 90.27%.

### 2.3. Statistical Analysis

The data analysis of this study was mainly carried out by SPSS25.0 (Chicago, IL, USA) and AMOS23.0 software (New York, NY, USA). Continuous variables were described by means of mean ±standard deviation or median ± interquartile range, whereas discontinuous variables were described as frequencies or percentages. The Kolmogorov–Smirnov test was used to analyze the normality of the distribution of variables. The normality test showed that all variables were skewed distribution. Therefore, Mann–Whitney U test, Wilcoxon W test (Kruskal–Wallis) and other non-parametric test methods for data analysis were used in this study. First, item analysis was performed on the scale, and an independent samples t-test was conducted on the subjects with the scores of the first 27% and the last 27% as the cut-off points for the high and low groups. Second, exploratory factor analysis (EFA) and confirmatory factor analysis (CFA) were performed to test the validity of the scale. Factor analysis is a statistical technique to extract common factors from groups of variables. The average score of vaccine literacy was calculated by dividing the total score of all participants by the number of participants. The test level was *p* value < 0.05.

### 2.4. Ethics

Ethical approval to conduct the study was obtained from the Institutional Review Board of College of Life Sciences, Central South University (Reference No.: 2022-1-22) and conducted following the guidelines of the Declaration of Helsinki. All participants were informed of the purpose of the study and volunteered to participate in the study, and all personal information of the participants used for the research was kept private.

## 3. Results

### 3.1. Item Analysis and Reliability and Validity

The results of the item analysis showed that all items reached a significance level of *p* < 0.001 for the high and low groups, with *t*-values > 3. All items were well differentiated and no items needed to be deleted.

SPSS25.0 was used to conduct exploratory factor analysis on all the samples. The results showed that Kaiser–Meyer–Olkin (KMO) value was 0.890, Bartlett’s test of sphericity was significant (χ^2^ = 2974.702, *p* < 0.001), indicating that all variables were correlated and the sample data were suitable for factor analysis. EFA was carried out by Varimax with Kaiser Normalization method. Three common factors with eigenvalues > 1 were extracted, whose eigenvalues were 6.035, 2.825 and 1.264, respectively. The cumulative rotation sum of squared loadings was 63.275%, and the factor loadings of all items were > 0.5. The scree plot showed that the data level off after the fourth principal component. Based on the above information, the first three principal components were initially extracted as common factors, respectively corresponding to the expected hypothesis dimensions: functional, interactive and critical vaccine literacy.

To further verify the validity of the scale, AMOS23.0 was used to conduct CFA on all samples. The convergent validity of the scale was tested by factor loading, composite reliability (CR), and average variance extracted (AVE) values. The results showed that, all items’ factor loadings were more than 0.5 except one item of functional vaccine literacy part. Therefore, it was decided to be deleted (original item 1: Did you find the material as a whole difficult to read or browse?). CFA was conducted again on the sample data. The results showed that the factor loading and the AVE values (0.571, 0.551 and 0.601) of the remaining items were all greater than 0.5, indicating that the revised scale showed good convergent validity.

The reliability of the scale was tested by Cronbach’s α, composite reliability (CR) and split-half reliability. The results confirmed that Cronbach’s α coefficients of the three dimensions were 0.838, 0.891 and 0.857, and CR were 0.841, 0.894 and 0.858, and the split-half coefficients were 0.818, 0.844 and 0.844, respectively, which were all greater than 0.8, indicating that the reliability of the scale was good.

To sum up, after reliability and validity test and item revision, the scale consisted of three dimensions with 15 items in total among which the functional vaccine literacy part has been adjusted from the original 5 items to 4 items, and the interactive and critical vaccine literacy parts consisted of 7 items and 4 items, respectively. Details of the results are shown in Table 1.

### 3.2. Sociodemographic Characteristics of Participants

The sociodemographic characteristics of the participants in this study are shown in Table 2. Nearly half of the participants were women (53.9%). Approximately 85.6% of the participants were aged between 18 and 45 years, with an average age of 34.49 ± 10.05 years. 45.6% of the respondents had a high school or below and 54.4% had a college degree or above. Most of the participants (69.6%) were urban residents, and approximately three-fifths (61.9%) were married. The occupational types of the participants were mainly concentrated in employees of enterprises and public institutions (30.2%), self-employed/freelancers (27.1%), students (18.5%) and farmers (12.4%). The average monthly income of a majority of participants (85.4 percent) was between 0 and 5,999 RMB. The vast majority of the respondents (98.9%) have been vaccinated against COVID-19, and most of them have completed the full course of vaccination (87.3%), while those who have not been vaccinated (1.1%) indicated that the main reasons for not vaccinating against COVID-19 were mainly contraindications to vaccination, such as pregnancy, having just given birth to a child, an allergy to the vaccine, etc.

### 3.3. COVID-19 Vaccine Literacy Scores

The participants had a mean level of COVID-19 VL of 3.71 ± 0.72 of which the level regarding the functional mean score was 4.41 ± 0.73, the minimum value was 1.25, and the maximum value was 5, while the average scores of interactive and critical vaccine literacy were 3.55 ± 0.95 and 3.28 ± 1.09, respectively, with minimum and maximum values of 1 and 5. The results indicated significant differences between functional, interactive, and critical vaccine literacy (*p* < 0.001), which was consistent with previous studies [42]. See Table 3 for details on the answers to each item.

### 3.4. The Relationship between Sociodemographic Characteristics and COVID-19 VL

**Figure 1:**
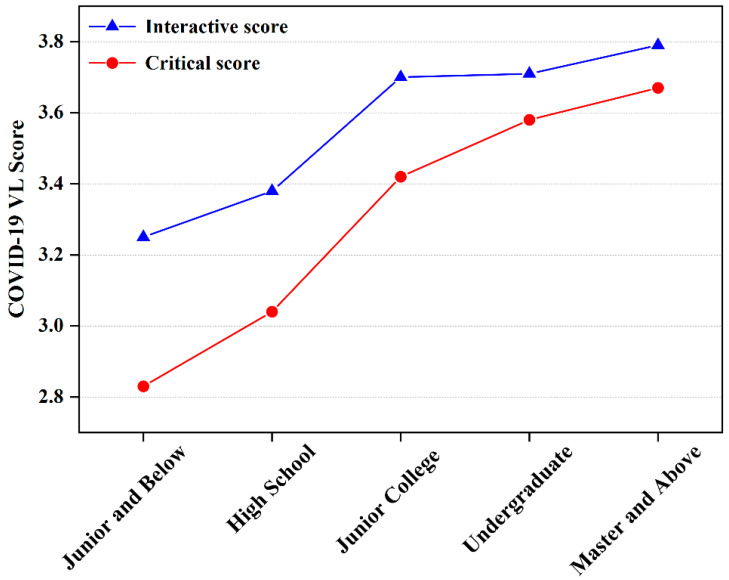
Relationship between different educational levels and vaccine literacy.

The associations between sociodemographic characteristics of participants and COVID-19 VL are shown in Table 4. Males had a significantly lower level of COVID-19 VL in comparison with females (*p* < 0.05). The level of COVID-19 VL regarding interactive (*p* = 0.001) and critical (*p* < 0.001) significantly increased with the level of education (Figure 1), which was confirmed in previous studies [35].

Significant associations were also found between functional vaccine literacy and age, marital status, and occupation, with the highest scores among those aged 55 years or older (4.79 ± 0.22), married (4.49 ± 0.66), career workers (4.65 ± 0.57), and retirees (4.65 ± 0.29). In addition, in terms of interactive vaccine literacy, there was a statistically relevant difference considering the average monthly income, with the highest scores among those with a monthly income of 9000–11,999 yuan (3.98 ± 0.86). Critical vaccine literacy was significantly associated with permanent residence and occupation, with urban residents (3.38 ± 1.07) and civil servants (4.06 ± 1.11) scoring the highest, while the associations between the presence or absence of vaccination against COVID-19, the process of COVID-19 vaccination and COVID-19 VL were not significantly.

### 3.5. Source of Information and Vaccination History

**Figure 2:**
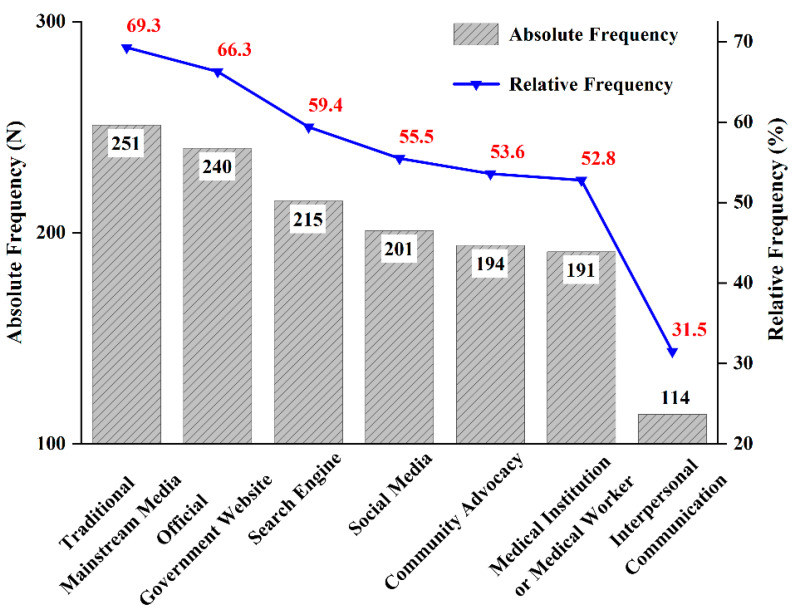
Participant’s choice of access to COVID-19 vaccine information.

Information sources of COVID-19 vaccine (Figure 2) most frequently used by the respondents were traditional mainstream media (69.3%), official government websites (66.3%), and search engines (59.4%), followed by social media (55.5%), community advocacy (53.6%), and medical institutions or medical workers (52.8%), while interpersonal communication channels accounted for less (31.5%).

Table 5 reports the relationship between COVID-19 VL and source of information. There was a significant association between the interactive vaccine literacy and the use of information sources. Participants who used traditional mainstream media, such as TV, radio, newspapers and periodicals (4.46 ± 0.68), official government websites, such as National Health Commission and Center for Disease Control and Prevention (CDC) (4.49 ± 0.69), and community advocacy (4.48 ± 0.70) scored significantly higher than those did not in functional vaccine literacy (*p* < 0.05). Regarding critical vaccine literacy, respondents who used official government websites (3.39 ± 1.07), community advocacy (3.73 ± 0.90), and social media (3.42 ± 1.06) scored higher compared with those who did not (*p* < 0.05). In terms of the number of information sources, pairwise comparisons showed that participants who used ≥5 information sources had higher vaccine literacy scores than those who used ≤2 sources (see Table S1).

In terms of other vaccination history (see Figure S1), most people had been vaccinated against Hepatitis B vaccine (60.8%), followed by Varicella (38.7%), Influenza (31.5%), Measles (30.1%), Rabies (28.7%), Diphtheria, Tetanus and Acellular Pertussis Combined Vaccine (DTP) (26.0%) and Tetanus (23.2%), respectively. HPV and other vaccines accounted for only 10.5% and 6.9%, respectively. Chinese residents were vaccinated with 2–3 kinds of vaccines per capita, and there was no statistically significant difference in COVID-19 VL among different numbers of other vaccines (*p* = 0.139).

## 4. Discussion

### 4.1. Main Findings

This study was the first to examine the validity and reliability of the Chinese version of the COVID-19 Vaccine Literacy Scale among Chinese adults. EFA and CFA analysis indicated that the Chinese COVID-19 Vaccine Literacy Scale consisted of three dimensions. Although different from previous studies divided into two dimensions of functional and interactive/critical vaccine literacy [29,34,44], the theoretical basis of these studies was the same, which were all based on the three-level model of health literacy proposed by Nutbeam [39]. There may be two possible reasons for the difference in dimensions: Firstly, the methods of factor extraction used in different studies were different. For example, Biasio [29] used PCA to investigate the vaccine literacy of Italian adults, while our study used factor analysis, which was more applicable to scenarios where the number of latent variables was unknown and the factors can be better explained through rotation techniques. Secondly, the cultural backgrounds and language habits of different countries were different. Although the translation strictly followed the work process, it was inevitable to ensure subtle differences in expression between different translations. In addition, the overall internal consistency coefficient of the Chinese version of COVID-19 Vaccine Literacy Scale was 0.885, which also confirmed the good internal consistency and stability of the scale compared with the Thai version (α = 0.81) and Croatian version (α = 0.81) of COVID-19 Vaccine literacy Scale.

In this study, we found that the overall COVID-19 VL of Chinese residents was at a medium level (mean = 3.71 ± 0.72). Consistent with previous studies [42], our study found that the score of functional vaccine literacy was higher than that of interactive and critical vaccine literacy, which could be interpreted as participants’ understanding of COVID-19 vaccine information was higher than their ability to acquire and judge.

In addition to exploring the applicability of the scale and assessing the overall VL level of the population, this study also analyzed the impact of sociodemographic variables on COVID-19 VL. In general, women showed higher vaccine literacy, which has been confirmed in similar previous studies [36], and previous studies on health literacy level assessment have also shown that men’s health literacy were significantly lower than women’s [45,46,47]. Compared with young people, the middle-aged and the elderly had higher functional vaccine literacy. The reason for this phenomenon may be that middle-aged and elderly people pay more attention to information about disease prevention and management due to their physiological needs, and therefore receive information about COVID-19 vaccine more frequently. Previous studies have also shown that age was also an important factor affecting the level of health literacy [28,48,49]. With increased access to medical services, the elderly may have a good level of knowledge on some basic health topics [50].

Compared with the low-educated population, the highly educated population performed a higher level of interactive and critical vaccine literacy. Similar conclusions were also drawn [36,46]. Improving education can increase people’s access to knowledge and behaviors to seek and understand health information [23]. In similar studies, education was seen as a determinant of people’s health literacy [46]. In addition to this, researchers have also found that educational attainment was an important factor in determining vaccine hesitancy [51,52]. Regarding marital status and permanent residence, this study observed lower functional vaccine literacy among unmarried and divorced or widowed persons compared to the married group, and those who lived in rural areas compared with urban residents had lower critical vaccine literacy, which is also a group of people who need to focus on vaccine advocacy and vaccination promotion activities in the future.

Regarding the relationship between occupational types and VL, there were significant differences in functional and critical vaccine literacy among different occupations, which was consistent with previous findings [29]. The functional VL of personnel of public institutions and retirees were the highest, while civil servants scored the highest in critical vaccine literacy. A possible explanation for this phenomenon was that in combination with the occupational background in China, the personnel of public institutions and civil servants were more likely to be frequently exposed to information about the national COVID-19 vaccination campaign and other information due to the particularity of their occupations, which made it easier to identify and verify false and erroneous information. Similar studies have also mentioned that employment was an important factor of health literacy level [48]. A survey study on the COVID-19 VL of the Croatian population showed that compared with the unemployed, the COVID-19 VL of employed persons were significantly lower [42], which was similar to our findings. As the authors explained, the reason why retired people had higher functional VL may be also the same as unemployed people, who had more time at home and can obtain a lot of news and information about the COVID-19 vaccine through various media compared to the employed [53].

By comparing vaccine literacy scores with or without using a certain information source, our study found that those who used official sources, such as the National Health Commission, the CDC, and community to obtain information about the COVID-19 vaccine had higher functional and critical vaccine literacy, possibly due to the higher professionalism and more rigorous wording of the information disseminated by this medium compared to the higher rate of misinformation in social media [54], which inspires us to increase the efforts of official media in vaccine knowledge dissemination. Nevertheless, we also observed the role of social media in the dissemination of vaccine information. The higher critical vaccine literacy of those who used social media to obtain vaccine information may be explained by the fact that despite the mixed information on the Internet, unlike official media, the open and interactive nature of social media platforms allowed people to analyze and compare the veracity of various information. Previous studies have also shown that social media interventions can strengthen COVID-19 immunization campaign [55].

Public media played an important role in guiding the public to follow the recommendations of health institutions regarding vaccination [56,57], however, in the era of proliferation of social media, groups represented by young people are more inclined to use online media to obtain information [58], and it is an issue worth considering how to regulate such media to fully play their role in vaccine knowledge popularization and promotion of vaccination. It has been shown that the sources of people’s access to vaccine information are characterized by multiple channels [59], which was also reflected in our study. In addition, our study observed that the level of vaccine literacy increased to some extent with the increase in the number of information sources, which may also indicate that the use of multiple information sources is more beneficial for people to compare the quality of information judged.

Unlike previous studies that mostly focused on assessing people’s functional vaccine literacy [60], our study adopted a more comprehensive indicators, adding two dimensions: interactive and critical vaccine literacy so that not only the respondents’ knowledge-based survey of information reading and comprehension could be addressed but the level of ability to acquire and judge information was also covered.

### 4.2. Limitations and Future Research

It is undeniable that this study still has some shortcomings. First of all, this survey was conducted in the form of an online questionnaire. The data sources were all self-assessed by the participants according to their own conditions. Although we had adopted strict procedures and measures to ensure the quality of the data as much as possible, it was still difficult to completely ensure that all the data were accurate. Second, since the original scale was compiled in an Italian context, the Chinese population’s understanding of the scale was inevitably influenced by the traditional Chinese culture, thus affecting the applicability of the scale in Chinese culture to some extent. However, in general, this was the first scale on China’s COVID-19 VL, which can bring some help to the evaluation of Chinese residents’ COVID-19 VL level. Finally, this study was a preliminary verification and exploration of the Chinese version of the scale, only to ensure that the sample size required for statistical analysis was met and larger-scale population studies have not been carried out. Future work can further expand the sample size for wider application and can compare COVID-19 VL among people with characteristics in different regions, provinces, and different ethnic groups so as to provide a clearer direction for promoting COVID-19 vaccination.

## 5. Conclusions

The results of this study indicate that the Chinese version of the COVID-19 Vaccine literacy Scale can be used as an effective and reliable tool to investigate the COVID-19 vaccine literacy level of Chinese residents. There is still room for improvement in the COVID-19 VL of the Chinese population. For nationwide vaccination, when implementing interventions, public health institutions should actively encourage the highly educated persons, employees of government agencies and public institutions to participate in COVID-19 vaccine knowledge popularization and other activities as the main force so as to drive people around them to actively participate in vaccination and prevent and control COVID-19. At the same time, relevant agencies should also focus on less educated persons, the population in rural areas and single people, who tend to have a low level of vaccine literacy. National health authorities are supposed to increase control over the quality of COVID-19 vaccine information on mass media and social media platforms to ensure that relevant information can be presented to the public in a timely and accurate manner.

## Figures and Tables

**Table 1:** Reliability and validity analysis.

Variable	Item	Factor Loading	CR	AVE	Cronbach’s α	Split-Half Reliability
Functional vaccine literacy	FVL1 ^1^	0.714	0.841	0.571	0.838	0.818
	FVL2 ^2^	0.848				
	FVL3 ^3^	0.759				
	FVL4 ^4^	0.693				
Interactive vaccine literacy	IVL1 ^5^	0.675	0.894	0.551	0.891	0.844
	IVL2 ^6^	0.716				
	IVL3 ^7^	0.776				
	IVL4 ^8^	0.830				
	IVL5 ^9^	0.841				
	IVL6 ^10^	0.740				
	IVL7 ^11^	0.584				
Critical vaccine literacy	CVL1 ^12^	0.757	0.858	0.601	0.857	0.844
	CVL2 ^13^	0.804				
	CVL3 ^14^	0.756				
	CVL4 ^15^	0.783				

^1^ FVL1: Did you find words in the material that you did not know? ^2^ FVL2: Did you find that the material was difficult to understand? ^3^ FVL3: Did you need much time to understand them? ^4^ FVL4: Did you or would you need someone to help you understand them? (e.g., asking others what the material means)? ^5^ IVL1: Did you know where to get information about the COVID-19 vaccine? ^6^ IVL2: Have you consulted more than one source of information? ^7^ IVL3: Did you or would you articulate the COVID-19 vaccine information you want? ^8^ IVL4: Did you find the COVID-19 vaccine information you are looking for? ^9^ IVL5: Did you understand the information you found about the COVID-19 vaccine? ^10^ IVL6: Have you had the opportunity to use the COVID-19 vaccine information? ^11^ IVL7: Did you discuss information about the COVID-19 vaccination with your doctor, family and friends? ^12^ CVL1: Did you consider whether the COVID-19 vaccine information collected was about your condition? ^13^ CVL2: Have you considered the credibility of the information sources regarding COVID-19 vaccines? ^14^ CVL3: Did you check whether the COVID-19 vaccine information was correct? ^15^ CVL4: Did you find any useful information to make a decision on whether or not to get COVID-19 vaccinated?

**Table 2:** Sociodemographic characteristics of the participants in this study.

Variable	Category	Number (n)	Percentage (%)
Gender	Male	167	46.1
	Female	195	53.9
Age	18–25	109	30.1
	26–35	80	22.1
	36–45	121	33.4
	46–55	45	12.4
	>55	7	1.9
Education	Junior and below	67	18.5
	High school	98	27.1
	Junior college	55	15.2
	Undergraduate	93	25.7
	Master and above	49	13.5
Permanent residence	Urban	252	69.6
	Rural	110	30.4
Marital status	Single	128	35.4
	Married	224	61.9
	Widowed or divorced	10	2.8
Occupation	Civil Servants	9	2.5
	Personnel of public institutions	44	12.2
	Employees of enterprises	65	18.0
	Self-employed/Freelance	98	27.1
	Farmers	45	12.4
	Students	67	18.5
	Retired	5	1.4
	Others	29	8.0
Average monthly income (RMB) ^1^	0–2999	173	47.8
	3000–5999	136	37.6
	6000–8999	30	8.3
	9000–11,999	14	3.9
	≥12,000	9	2.5
COVID-19 vaccination status	The first injection completed (Booster injection uncompleted)	7	1.9
	The first and second injections completed (booster injections uncompleted)	35	9.7
	Booster injections completed ^2^	112	30.9
	Full vaccination has been completed ^3^	204	56.4
	Not yet vaccinated against COVID-19	4	1.1

^1^ Average monthly income: It refers to a person’s average monthly salary, which is equal to one year’s income divided by 12 months; ^2^ Booster injections completed: It refers to participants who chose to receive either one or two shots of COVID-19 vaccine in the first round; ^3^ Full vaccination has been completed: It refers to participants who chose to receive three doses of COVID-19 vaccine in the first round.

**Table 3:** Responses to each item of the COVID-19 Vaccine Literacy Scale.

Variable	Item	N (%)					M (SD)	Min	Max
		Never	Rarely	Sometimes	Often	Always			
Functional vaccine literacy	FVL2	186 (51.4)	91 (25.1)	68 (18.8)	14 (3.9)	3 (0.8)	4.22 (0.94)	1	5
	FVL3	231 (63.8)	74 (20.4)	42 (11.6)	11 (3.0)	4 (1.1)	4.43 (0.89)	1	5
	FVL4	228 (63.0)	75 (20.7)	44 (12.2)	7 (1.9)	8 (2.2)	4.40 (0.93)	1	5
	FVL5	265 (73.2)	53 (14.6)	35 (9.7)	5 (1.4)	4 (1.1)	4.57 (0.81)	1	5
Interactive vaccine literacy	IVL1	52 (14.4)	34 (9.4)	54 (14.9)	120 (33.1)	102 (28.2)	3.51 (1.37)	1	5
	IVL2	28 (7.7)	39 (10.8)	63 (17.4)	146 (40.3)	86 (23.8)	3.62 (1.18)	1	5
	IVL3	44 (12.2)	44 (12.2)	66 (18.2)	122 (33.7)	86 (23.8)	3.45 (1.30)	1	5
	IVL4	25 (6.9)	37 (10.2)	56 (15.5)	142 (39.2)	102 (28.2)	3.72 (1.18)	1	5
	IVL5	23 (6.4)	26 (7.2)	72 (19.9)	140 (38.7)	101 (27.9)	3.75 (1.13)	1	5
	IVL6	27 (7.5)	52 (7.5)	84 (23.2)	127 (35.1)	72 (19.9)	3.46 (1.18)	1	5
	IVL7	30 (8.3)	56 (15.5)	102 (28.2)	116 (32.0)	58 (16.0)	3.32 (1.16)	1	5
Critical vaccine literacy	CVL1	42 (11.6)	61 (16.9)	91 (25.1)	97 (26.8)	71 (19.6)	3.26 (1.27)	1	5
	CVL2	55 (15.2)	49 (13.5)	91 (25.1)	90 (24.9)	77 (21.3)	3.23 (1.34)	1	5
	CVL3	56 (15.5)	47 (13.0)	89 (24.6)	102 (28.2)	68 (18.8)	3.22 (1.32)	1	5
	CVL4	47 (13.0)	38 (10.5)	77 (21.3)	117 (32.3)	83 (22.9)	3.42 (1.30)	1	5

**Table 4:** Relationship between sociodemographic characteristics and COVID-19 vaccine literacy.

Variable	Category	VL Functional Score		VL Interactive Score		VL Critical Score		VL Score	
		Mean ± SD	*p* Value	Mean ± SD	*p* Value	Mean ± SD	*p* Value	Mean ± SD	*p* Value
Gender	Male	4.25 ± 0.80	**<0.001** ^1^	3.38 ± 0.97	**0.002**	3.09 ± 1.08	**0.002**	3.53 ± 0.74	**<0.001**
	Female	4.55 ± 0.65		3.69 ± 0.90		3.45 ± 1.08		3.85 ± 0.67	
Age	18–25	4.28 ± 0.82	**0.049**	3.64 ± 0.93	0.241	3.44 ± 0.92	0.164	3.76 ± 0.67	0.324
	26–35	4.45 ± 0.64		3.45 ± 0.93		3.37 ± 1.06		3.70 ± 0.69	
	36–45	4.42 ± 0.75		3.44 ± 0.98		3.09 ± 1.21		3.61 ± 0.77	
	46–55	4.58 ± 0.63		3.71 ± 0.92		3.19 ± 1.18		3.80 ± 0.78	
	>55	4.79 ± 0.22		3.78 ± 0.79		3.64 ± 1.11		4.01 ± 0.61	
Education	Junior and below	4.32 ± 0.82	0.093	3.25 ± 0.96	**0.001**	2.83 ± 1.13	**<0.001**	3.42 ± 0.78	**<0.001**
	High school	4.52 ± 0.68		3.38 ± 0.93		3.04 ± 1.04		3.59 ± 0.65	
	Junior college	4.34 ± 0.81		3.70 ± 0.95		3.42 ± 1.15		3.80 ± 0.77	
	Undergraduate	4.33 ± 0.73		3.71 ± 0.94		3.58 ± 0.97		3.84 ± 0.70	
	Master and above	4.53 ± 0.58		3.79 ± 0.84		3.67 ± 1.02		3.95 ± 0.63	
Permanent residence	Rural	4.48 ± 0.73	0.065	3.44 ± 0.99	0.184	3.05 ± 1.13	**0.010**	3.62 ± 0.76	0.172
	Urban	4.37 ± 0.73		3.59 ± 0.92		3.38 ± 1.07		3.74 ± 0.70	
Marital status	Single	4.27 ± 0.80	**0.005**	3.65 ± 0.91	0.259	3.43 ± 0.93	0.250	3.76 ± 0.66	0.730
	Married	4.49 ± 0.66		3.49 ± 0.96		3.21 ± 1.16		3.68 ± 0.74	
	Widowed or divorced	4.30 ± 1.17		3.47 ± 1.06		3.00 ± 1.40		3.57 ± 1.00	
Occupation	Civil Servants	3.94 ± 0.91	**0.012**	4.10 ± 0.92	0.051	4.06 ± 1.11	**0.007**	4.04 ± 0.90	0.057
	Personnel of public institutions	4.65 ± 0.57		3.64 ± 0.99		3.50 ± 1.13		3.87 ± 0.72	
	Employees of enterprises	4.35 ± 0.73		3.58 ± 0.88		3.48 ± 0.94		3.76 ± 0.66	
	Self-employed/Freelance	4.45 ± 0.71		3.33 ± 0.98		3.02 ± 1.14		3.54 ± 0.74	
	Farmers	4.43 ± 0.78		3.38 ± 1.00		2.89 ± 1.17		3.53 ± 0.81	
	Students	4.22 ± 0.84		3.68 ± 0.87		3.49 ± 0.86		3.78 ± 0.62	
	Retired	4.65 ± 0.29		3.80 ± 0.82		3.15 ± 1.10		3.85 ± 0.60	
	Others	4.52 ± 0.56		3.78 ± 0.91		3.34 ± 1.26		3.86 ± 0.73	
Average monthly income (RMB)	0–2999	4.43 ± 0.75	0.852	3.59 ± 0.91	**0.041**	3.26 ± 1.02	0.095	3.73 ± 0.68	0.108
	3000–5999	4.41 ± 0.70		3.41 ± 1.00		3.19 ± 1.18		3.62 ± 0.77	
	6000–8999	4.36 ± 0.72		3.81 ± 0.82		3.78 ± 0.92		3.95 ± 0.65	
	9000–11,999	4.41 ± 0.68		3.98 ± 0.86		3.30 ± 1.31		3.91 ± 0.77	
	≥12,000	4.03 ± 1.13		3.21 ± 1.03		3.36 ± 1.18		3.47 ± 0.76	
Whether to get the COVID-19 vaccine	Yes	4.41 ± 0.73	0.488	3.54 ± 0.95	0.799	3.28 ± 1.10	0.473	3.70 ± 0.72	0.836
	No	4.19 ± 0.80		3.79 ± 0.72		3.69 ± 0.63		3.87 ± 0.64	
COVID-19 vaccination status	The first injection completed (Booster injection uncompleted)	4.46 ± 0.81	0.440	3.14 ± 0.87	0.194	2.93 ± 1.03	0.108	3.44 ± 0.66	0.139
	The first and second injections completed (Booster injections uncompleted)	4.28 ± 0.82		3.34 ± 1.05		3.09 ± 1.25		3.53 ± 0.84	
	Booster injections completed ^2^	4.42 ± 0.65		3.67 ± 0.93		3.49 ± 1.00		3.82 ± 0.69	
	Full vaccination has been completed ^3^	4.43 ± 0.76		3.52 ± 0.94		3.21 ± 1.11		3.68 ± 0.72	

^1^ Bold: It means that *p* value is less than 0.05, indicating there is a statistical significant difference; ^2^ Booster injections completed: It refers to participants who chose to receive either one or two shots of COVID-19 vaccine in the first round; ^3^ Full vaccination has been completed: It refers to participants who chose to receive three doses of COVID-19 vaccine in the first round.

**Table 5:** Relationship between information sources and COVID-19 vaccine literacy.

Category		N	VL Functional Score		VL Interactive Score		VL Critical Score		VL Score	
			Mean ± SD	*p* Value	Mean ± SD	*p* Value	Mean ± SD	*p* Value	Mean ± SD	*p* Value
Traditional mainstream media	Yes	251	4.46 ± 0.68	**0.028** ^1^	3.60 ± 0.95	**0.035**	3.33 ± 1.05	0.274	3.76 ± 0.69	**0.034**
	No	111	4.28 ± 0.83		3.41 ± 0.93		3.18 ± 1.19		3.58 ± 0.78	
Official government websites	Yes	240	4.49 ± 0.69	**0.003**	3.69 ± 0.92	**<0.001**	3.39 ± 1.07	**0.014**	3.82 ± 0.69	**<0.001**
	No	122	4.25 ± 0.79		3.25 ± 0.93		3.08 ± 1.11		3.47 ± 0.73	
Community advocacy	Yes	194	4.48 ± 0.70	**0.025**	3.73 ± 0.90	**<0.001**	3.41 ± 1.11	**0.013**	3.84 ± 0.70	**<0.001**
	No	168	4.32 ± 0.76		3.33 ± 0.96		3.13 ± 1.07		3.54 ± 0.72	
Interpersonal communication	Yes	114	4.39 ± 0.80	0.601	3.73 ± 0.96	**0.006**	3.41 ± 1.13	0.069	3.82 ± 0.74	**0.033**
	No	248	4.42 ± 0.70		3.46 ± 0.93		3.22 ± 1.08		3.65 ± 0.71	
Medical Institution or Medical Worker	Yes	191	4.45 ± 0.70	0.281	3.69 ± 0.90	**0.001**	3.38 ± 1.05	0.060	3.81 ± 0.68	**0.004**
	No	171	4.36 ± 0.77		3.38 ± 0.97		3.17 ± 1.13		3.59 ± 0.75	
Social media	Yes	201	4.45 ± 0.70	0.268	3.72 ± 0.91	**<0.001**	3.42 ± 1.06	**0.004**	3.84 ± 0.71	**<0.001**
	No	161	4.36 ± 0.77		3.32 ± 0.94		3.11 ± 1.12		3.54 ± 0.71	
Search engine	Yes	215	4.43 ± 0.73	0.273	3.65 ± 0.91	**0.019**	3.34 ± 1.07	0.234	3.77 ± 0.71	**0.047**
	No	147	4.38 ± 0.74		3.40 ± 0.98		3.20 ± 1.12		3.61 ± 0.74	

^1^ Bold: It means that *p* value is less than 0.05, indicating there is a statistical significant difference.

## Data Availability

The data that support the findings of this study are available from the corresponding author upon reasonable request.

## Supplementary Materials

The following supporting information can be downloaded at: https://www.mdpi.com/article/10.3390/ijerph192013601/s1.

## Appendix A

**Table A1:** The initial Chinese version of the COVID-19 vaccine literacy scale.

Variable	Items	Measurement (Score)
VL functional skills	When reading or listening to information about COVID-19 vaccines:	5 points Likert scale: 5-none; 4-less; 3-once in a while; 2-often; 1-Always
	1. Did you find the material as a whole difficult to read or browse?	
	2. Did you find words in the material that you did not know?	
	3. Did you find that the material was difficult to understand?	
	4. Did you need much time to understand them?	
	5. Did you or would you need someone to help you understand them? (e.g., asking others what the material means)	
VL interactive skills	When looking for information about COVID-19 vaccines:	5 points Likert scale: 5-none; 4-less; 3-once in a while; 2-often; 1-Always
	1. Did you know where to get information about the COVID-19 vaccine?	
	2. Have you consulted more than one source of information?	
	3. Did you or would you specify the vaccine information you want?	
	4. Did you find the COVID-19 vaccine information you are looking for?	
	5. Did you understand the information you found about the COVID-19 vaccine?	
	6. Have you had the opportunity to use the COVID-19 vaccine information?	
	7. Did you discuss information about the COVID-19 vaccination with your doctor, family and friends?	
VL critical skills	When looking for information about COVID-19 vaccines:	5 points Likert scale: 5-none; 4-less; 3-once in a while; 2-often; 1-Always
	1. Did you consider whether the COVID-19 vaccine information collected was about your condition?	
	2. Have you considered the credibility of the information sources regarding COVID-19 vac-cines?	
	3. Did you check whether the COVID-19 vaccine information was correct?	
	4. Did you find any useful information to make a decision on whether or not to get COVID-19 vaccinated?	
