# Supplementary material for: The Development and Preliminary Application of the Chinese Version of the COVID-19 Vaccine Literacy Scale
Source: Int J Environ Res Public Health. 2022 Oct 20;19(20):13601. doi: 10.3390/ijerph192013601 (PMC9603366; doi:10.3390/ijerph192013601)
Supplement: Supplementary file 1 [file ijerph-19-13601-s001.zip › ijerph-1897754-supplementary.pdf]

Table S1: Comparison of COVID-19 vaccine literacy among different number of information sources

Sample1-Sample2	Number of information sources	Mean Rank	COVID-19 VL (Mean ± SD)	P value
2-5	2	127.48	3.35±0.65	0.010
	5	202.24	3.84±0.68	
2-6	2	127.48	3.35±0.65	< 0.001
	6	244.38	4.11±0.75	
2-7	2	127.48	3.35±0.65	0.002
	7	205.63	3.88±0.69	
1-6	1	155.61	3.51±0.83	0.002
	6	244.38	4.11±0.75	

Only the data with significant differences are listed in the table

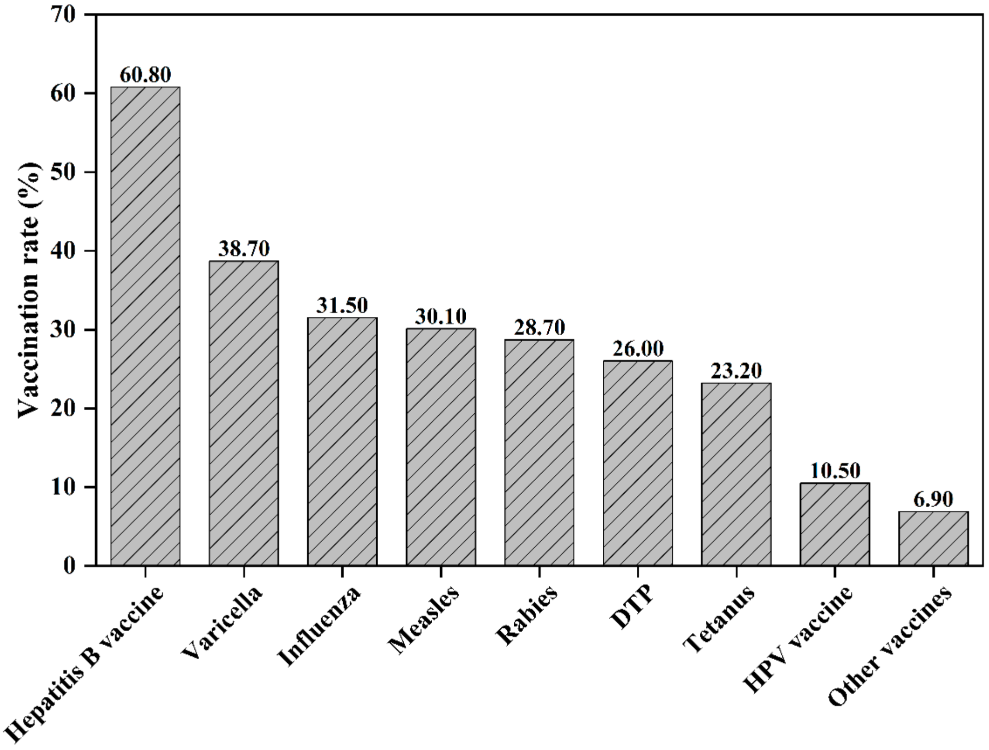

Figure S1: Participants' other vaccination history
